# Supplementary material for: Machine learning inference of continuous single-cell state transitions during myoblast differentiation and fusion
Source: Mol Syst Biol. 2024 Jan 18;20(3):217–41. doi: 10.1038/s44320-024-00010-3 (PMC10912675; doi:10.1038/s44320-024-00010-3)
Supplement: Supplementary file 1 — Appendix [file 44320_2024_10_MOESM1_ESM.docx]

APPENDIX
**Machine learning inference of continuous single-cell state transitions during myoblast differentiation and fusion**

**Table of Contents**

| Appendix Figure S1 | Page 1 |
| --- | --- |
| Appendix Figure S2 | Page 2 |
| Appendix Figure S3 | Page 3-4 |
| Appendix Figure S4 | 5-6 |
| Appendix Figure S5 | 7 |
| Appendix Figure S6 | 8 |
| Appendix Figure S7 | 9 |
| Appendix Figure S8 | 10-11 |
| Appendix Figure S9 | 12 |
| Appendix Figure S10 | 13-14 |
| Appendix Figure S11 | 15 |
| Appendix Figure S12 | 16 |
| Appendix Figure S13 | 17 |
| Appendix Figure S14 | 18-19 |
| Appendix Figure S15 | 20-21 |
| Appendix Figure S16 | 22 |
| Appendix Figure S17 | 23 |
|  |  |

Appendix Figure S1


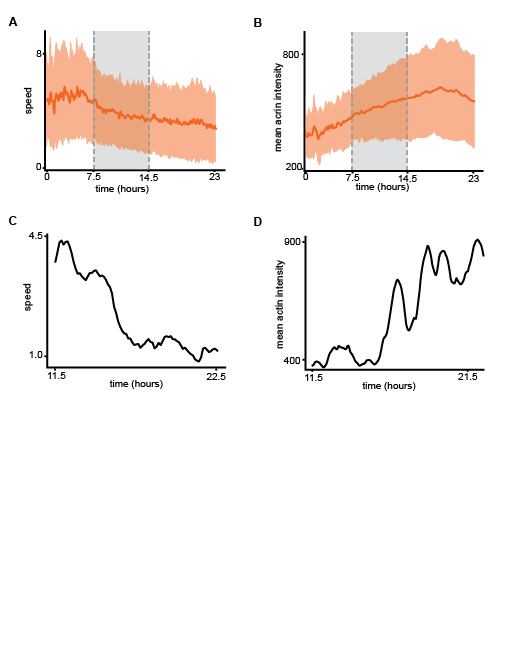


**Appendix Figure S1 Dynamic changes in motility and actin intensity measured in semi-manually tracked single cells**(**A-B**) Mean (line) and standard deviation (shade) of single cell speed (A) and mean actin intensity (B) over time during differentiation (ERKi; N = 575 cells). (**C-D**) Representative single cell’s speed (C) and mean actin intensity (D) trajectories.

Appendix Figure S2


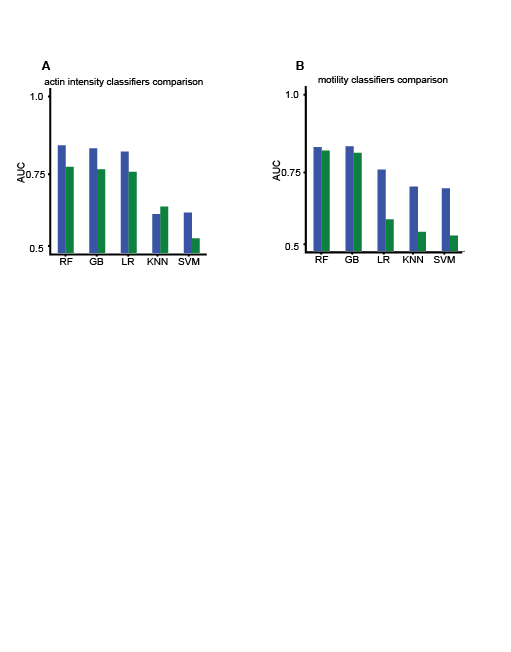


Appendix Figure S2 Comparison of classification algorithms

(**A-B**) Area under the receiver operating characteristic (ROC) curve (AUC) for classifiers trained with actin intensity (A) and motility (B) time series, using random forest (RF), gradient boosting (GB), logistic regression (LR), k-nearest neighbors (KNN), and support vector machines (SVM). Blue/green – flipped train/test experiments (blue: 789 cells; green: 736 cells). Average AUCs for actin intensity classifiers were 0.78 (RF), 0.77 (GB), 0.76 (LR), 0.59 (KNN), 0.54 (SVM). Average AUCs for motility classifiers were 0.8 (RF), 0.8 (GB), 0.64 (LR), 0.59 (KNN), 0.59 (SVM).

Appendix Figure S3


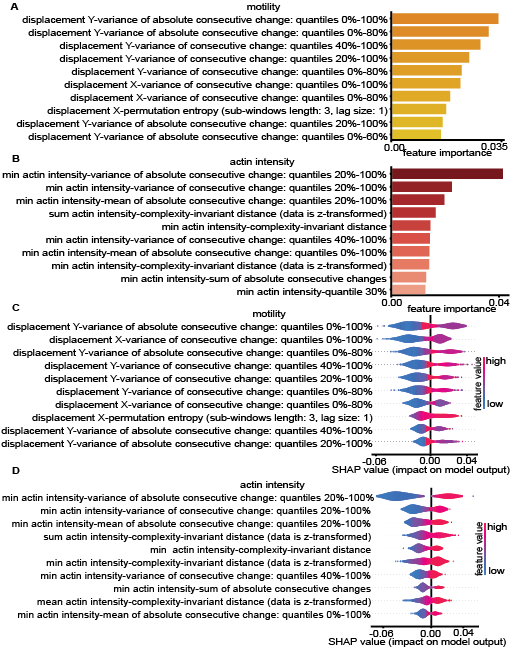


Appendix Figure S3 Random forests feature importance

Top ten features most relevant for differentiation classification using random forest classifiers’ importance (A-B) and SHapley Additive exPlanation (SHAP) (C-D). Both approaches find very similar important features: variance of consecutive change in displacement, permutation entropy of displacement, variance of consecutive change in actin intensity, complexity-invariant distance of actin intensity and sum of absolute consecutive changes in actin intensity. **(A-B)** Feature importance of random forest classifiers trained to discriminate undifferentiated/differentiated cell states, on features extracted from motility (yellow) and actin intensity (red), time series using “tsfresh” package. The 10 most important features are shown. **(C-D)** SHAP summary plots of motility (C) and actin intensity (D) classifiers, produced by the SHAP python package (Lundberg & Lee, 2017). The plot illustrates the feature relevance and combines feature attributions to the model’s predictive performance. Color is dependent on the feature values. The 10 most important features are shown.

Appendix Figure S4


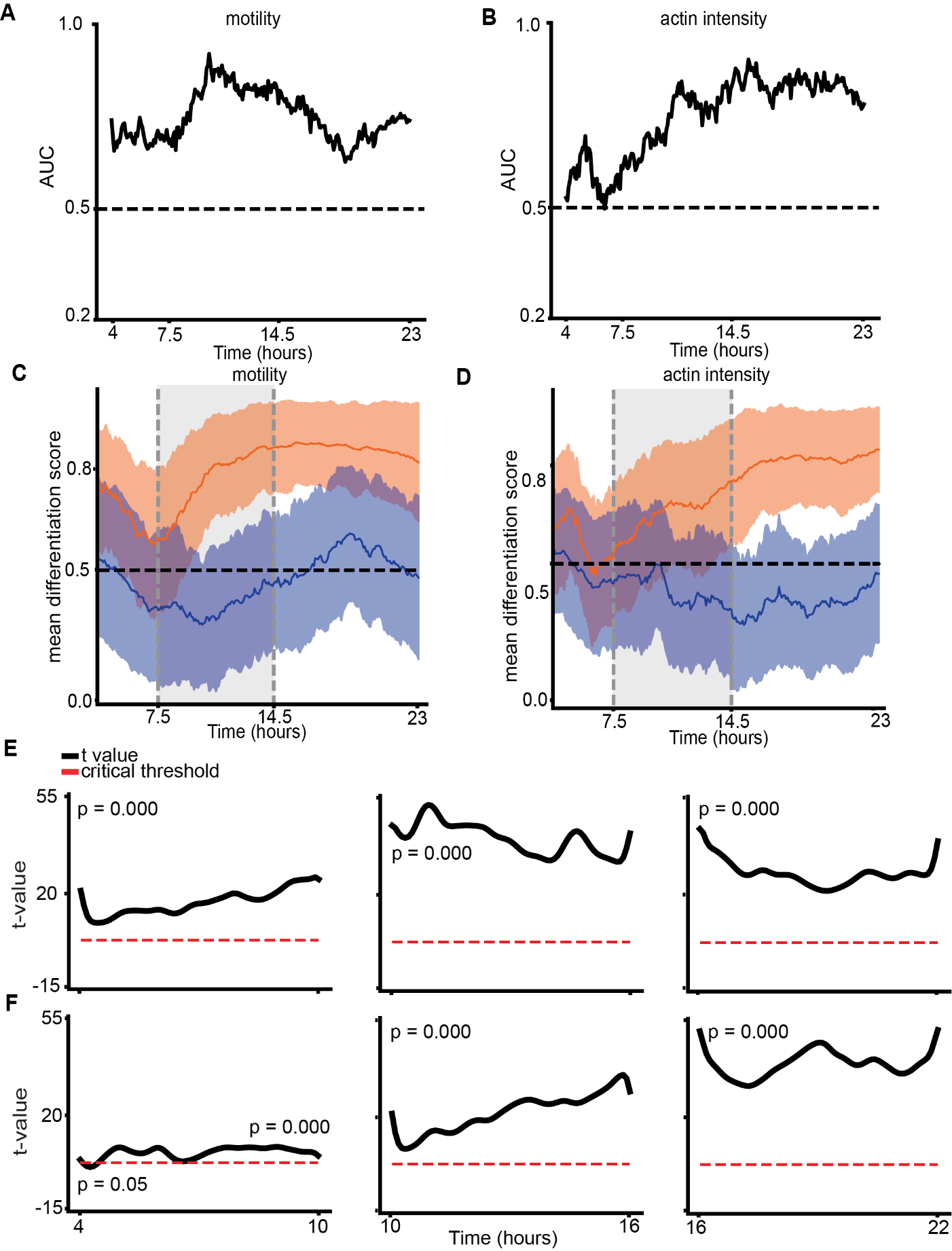


Appendix Figure S4 Inference of differentiation trajectories - flipped experiments for train/test

**(A-B)** Classification performance over time for the entire experiment. Area under the receiver operating characteristic (ROC) curve (AUC) over time for classifiers trained with motility (B) and actin intensity (C) time-series. The AUC was calculated for 736 cells from an independent experiment. Classification performance of a random model (AUC = 0.5) is marked with a dashed horizontal line. Compare with Fig. 2B-C. **(C-D)** Mean (solid line) and standard deviation (shade) of the differentiation score over time of ERKi- (orange) and DMSO- (blue) treated cells using the motility (B) and the actin intensity (C) classifiers over time for the entire experiment (ERKi: 420 cells; DMSO: 249 cells). Dashed vertical gray rectangle highlights the time interval of 7.5-14.5 hours, where both models predicted the differentiation occurs. Compare with Fig. S21. **(E-F)** Random Field Theory (RFT) analysis of the differences in differentiation scores between cells treated with ERKi and cells treated with DMSO, using the motility (E) and actin intensity (F) models (ERKi: 420 cells; DMSO: 249 cells). The black line represents the results of a two-sample t-test conducted over the duration of the experiment. Red dotted horizontal lines indicate the critical RFT threshold at α=0.05. The depicted p-values denote the probability that trajectories within those temporal extents would surpass the critical RFT threshold (refer to Methods for details). A statistically significant difference in differentiation scores is observed at all time intervals (motility), and from the 6.5 hour (actin intensity).

Appendix Figure S5


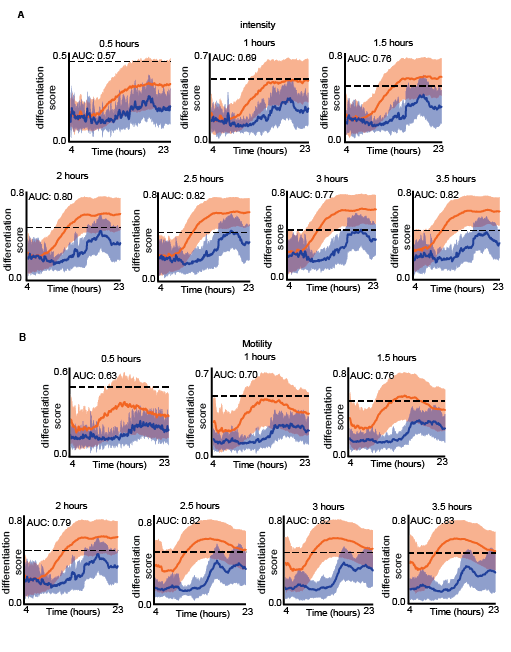


Appendix Figure S5 Classification sensitivity analysis: temporal segment size

**(A-B)** Mean (solid line) and standard deviation (shade) of the differentiation score over time of ERKi- (orange) and DMSO- (blue) treated cells using the motility (B) and the actin intensity (C) classifiers, trained with different sizes of temporal segment. Temporal segment’s size (in hours) is shown above each graph. Corresponding AUCs are reported in the figure. The temporal segment size for both classifiers was 2.5 hours (ERK: 575 cells; DMSO: 103 cells).

Appendix Figure S6


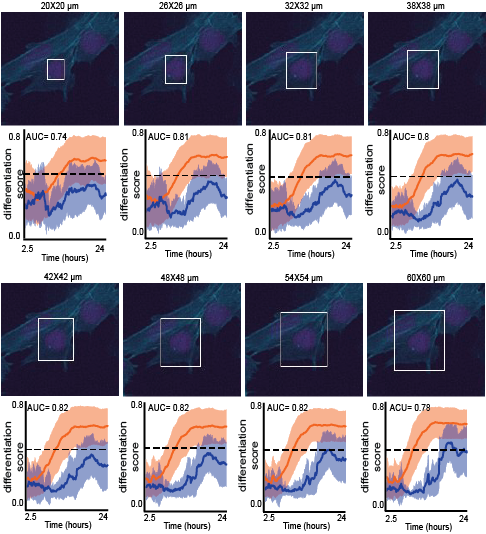


Appendix Figure S6 Classification sensitivity analysis: actin intensity quantification window size

Top: an illustration of the actin quantification window size. Bottom: Mean (solid line) and standard deviation (shade) of the differentiation score over time of ERKi- (orange) and DMSO- (blue) treated cells using the actin intensity classifier, trained using these window sizes. Corresponding AUCs are reported in the figure (ERK: 575 cells; DMSO: 103 cells).

Appendix Figure S7


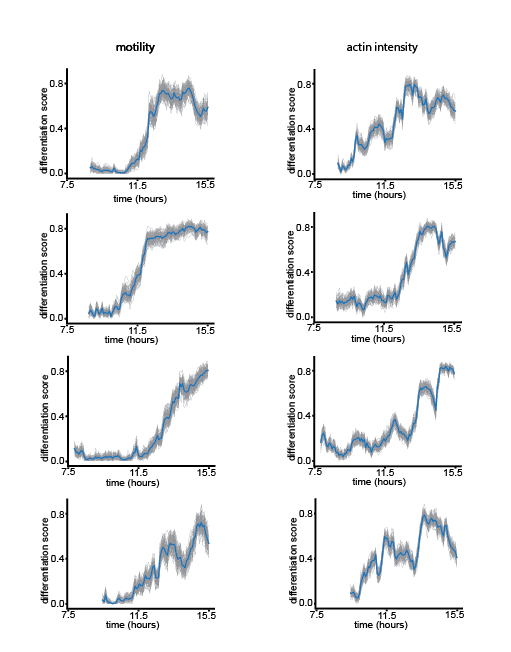


Appendix Figure S7 Prediction interval of single cells differentiation trajectories shows robustness across multiple model training.

Mean differentiation score (blue line) and differentiation trajectories (gray lines) predicted by 100 independent models for both motility (left) and actin intensity (right) models. The figure shows the prediction interval of representative single cells differentiation trajectories.

Appendix Figure S8


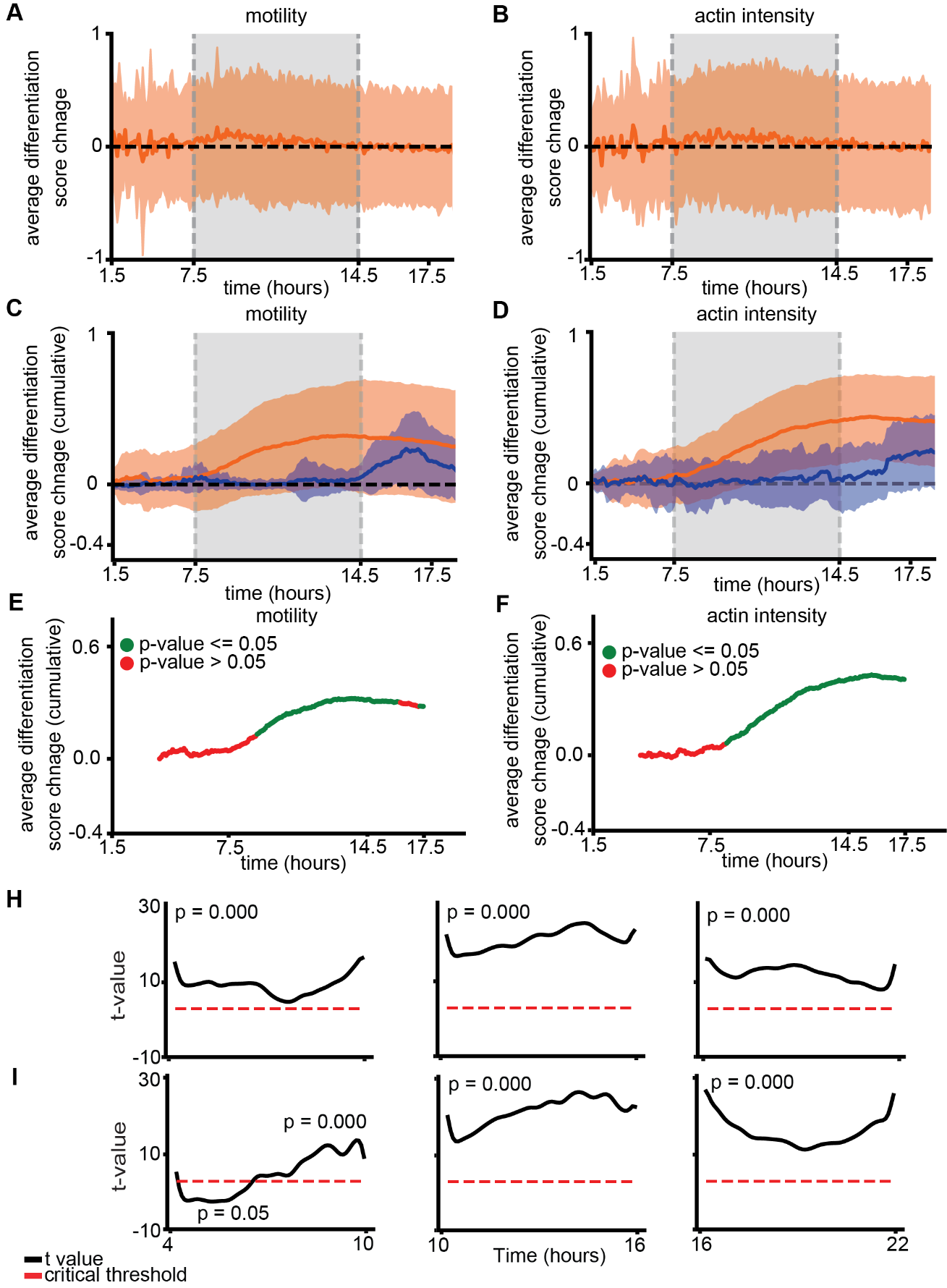


Appendix Figure S8 Differentiation scores’ temporal derivative and integration over time

**(A-B)** Mean (solid line) and standard deviation (shade) of temporal change in differentiation score for motility (A) and actin intensity (B) models (ERK: 575 cells; DMSO: 103 cells). **(C-D)** Mean (solid line) and standard deviation (shade) of the cumulative change in differentiation score for motility. Dashed vertical gray rectangle highlights the time interval of 7.5-14.5 hours, where both models predicted the differentiation occurs. (C) and actin intensity (D) models. Dashed vertical gray rectangle highlights the time interval of 7.5-14.5 hours, where both models predicted the differentiation occurs (ERK: 575 cells; DMSO: 103 cells). **(E-F)** Average cumulative change in differentiation score over time of Erki treated cells, for motility **(E)** and actin intensity models **(F)**. P-values resulting from a t-test between the cumulative change in differentiation score of cells treated with Erki and DMSO are demonstrating significant (green, p-value <= 0.05) and non-significant (red, p-value > 0.05) difference (ERK: 575 cells; DMSO: 103 cells). **(H-I)** Random Field Theory (RFT) analysis of the differences in differentiation scores between cells treated with ERKi and cells treated with DMSO, using the motility (H) and actin intensity (I) models (ERKi: 575 cells; DMSO: 103 cells). The black line represents the results of a two-sample t-test conducted over the duration of the experiment. Red dotted horizontal lines indicate the critical RFT threshold at α=0.05. The depicted p-values denote the probability that trajectories within those temporal extents would surpass the critical RFT threshold (refer to Methods for details). A statistically significant difference in differentiation scores is observed at all time intervals (motility), and from the 6th hour (actin intensity).

Appendix Figure S9


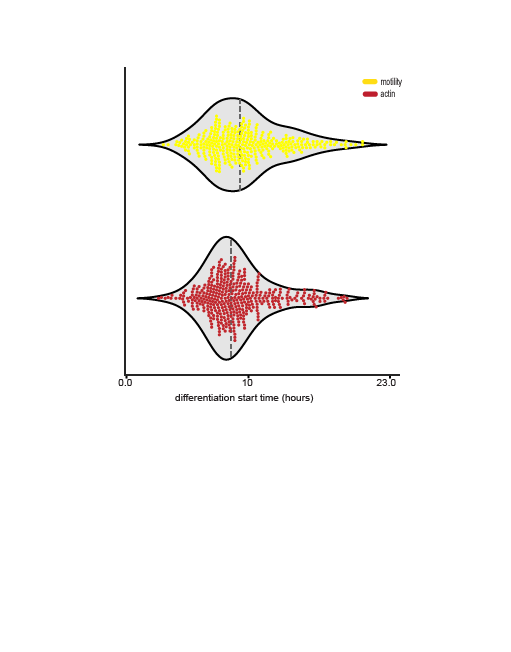


Appendix Figure S9 Predicted onset of differentiation is heterogeneous

Distribution of single cells predicted onset of differentiation state transition as predicted by the motility (yellow) and the actin intensity (red) models. N = 343 (motility) and N = 390 (actin intensity) cells were included in the calculation according to our definition of the onset (see methods). Median start times were 9.42 hours (motility) and 8.67 hours (actin intensity).

Appendix Figure S10


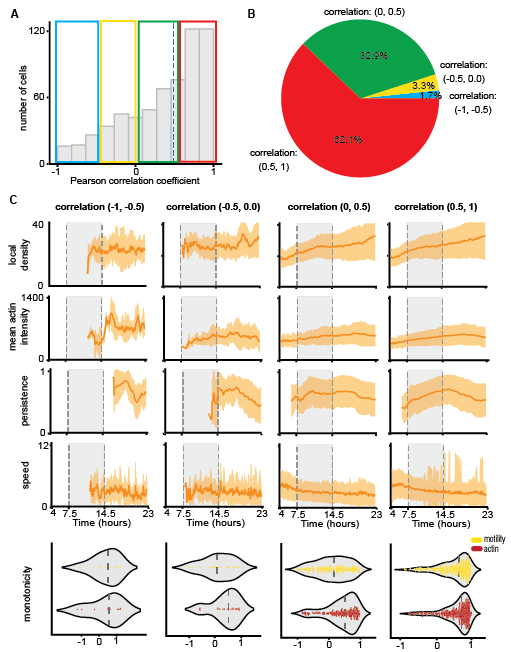


Appendix Figure S10. Motility and actin intensity models agree on monotonically increasing differentiation trajectories

**(A)** Distribution of single cells agreement between the predictions of motility and actin intensity classifiers, determined by the Pearson correlation coefficient of the correlation between the inferred trajectories in the differentiation time interval of 7.5-14.5 hours. The agreement was assessed for 575 ERKi-treated cells and 103 DMSO-treated cells. The Median Pearson correlation coefficient (green dashed line) was 0.48. Colored rectangles mark cells with a correlation coefficient range of [-1, -0.5] (blue, 10 cells), [-0.5, 0] (yellow, 20 cells) [0, 0.5] (green, 198 cells), and [0.5, 1] (red, 374 cells). **(B)** Pie chart representing single cell population fractions in different correlation coefficients ranges, corresponding to the colored rectangles described above. **(C)** Cellular properties analysis of cells sub-groups, partitioned by the agreement rate between the predictions of the motility and actin intensity models. Top: mean (solid line) and standard deviation (shade) of cellular properties measured over time: local density, mean actin intensity, persistence, and speed. Bottom: monotonicity rate, as defined by the Spearman correlation coefficient between differentiation score and time at the time interval of 7.5-14.5. Median monotonicity rates were 0.43 (motility) and 0.482 (actin) for range [-1, -0.5] (N=10 cells), 0.06 (motility) and 0.5 (actin) for range [-0.5, 0] (N=20 cells), 0.11 (motility) and 0.53 (actin) for range [0, 0.5] (N=198 cells), and 0.66 (motility) and 0.72 (actin) for range [-1, -0.5] (N=374 cells).

Appendix Figure S11


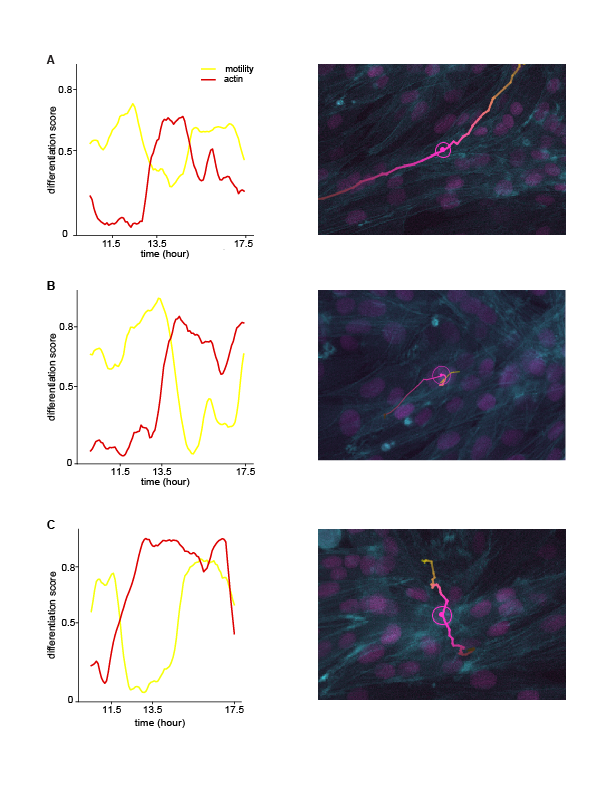


Appendix Figure S11: Exploration of disagreement between the motility and actin models.

Left: representative single cells differentiation scores predicted using the motility (yellow) and actin (red) models. Right: representative snapshots of these cells at time points where the models highly disagree. **(A)** The examined single cell is barely visible. Thus, actin intensity measurements consist of other cells rather than the examined one. The Pearson correlation coefficient was -0.91. **(B)** The examined cell crawled below another cell at several time points, causing bias in the actin intensity measurements. The Pearson correlation coefficient was -0.23. **(C)** The examined cell entered a crowded region, thus the intensity measurements represent other cells. The Pearson correlation coefficient was -0.91.

Appendix Figure S12


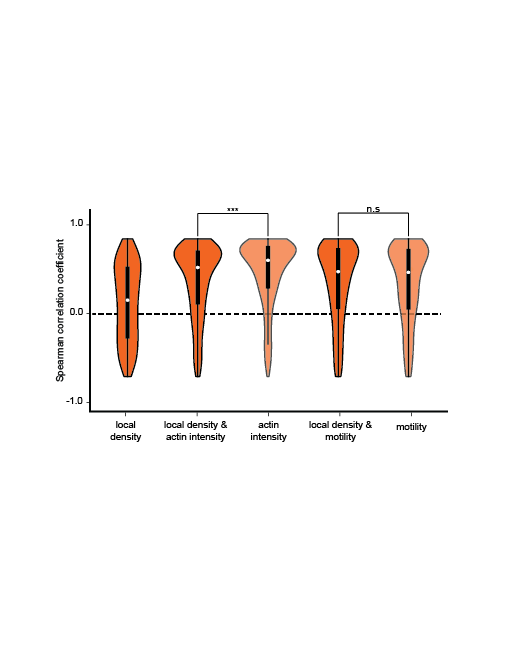


Appendix Figure S12 Local density does not improve quantification of the continuous differentiation state

Distribution of single cell correlation between the differentiation score and time for classifiers trained using features that include or exclude local density. Dashed horizontal line shows no correlation. Median values (shown in white) were 0.09 (local density), 0.62 (local density + actin intensity), 0.67 (actin intensity), 0.53 (local density + motility), 0.53 (motility). The correlations of the actin intensity classifier were higher without the local density feature (Wilcoxon rank sign test p-value = 5.7 * 10^-10^), the correlations of the motility classifier were not improved by including the local density feature. N = 575 cells. *** - p-value < 0.0001, n.s – not significant.

Appendix Figure S13


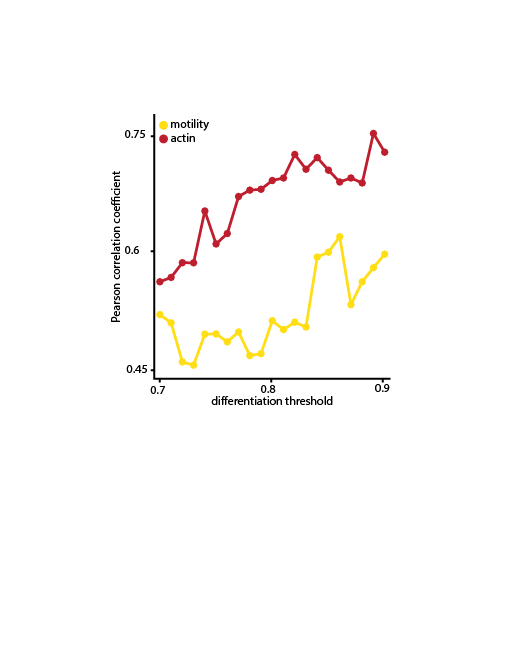


Figure S13 Sensitivity analysis: threshold for terminal differentiation

Pearson correlation coefficient of the correlation between the (predicted) terminal differentiation time and the manually annotated fusion time, for different terminal differentiation thresholds. The number of cells that are identified as terminally differentiated depends on the threshold, thus as the threshold increases- the number of identified cells decreases.

Appendix Figure S14


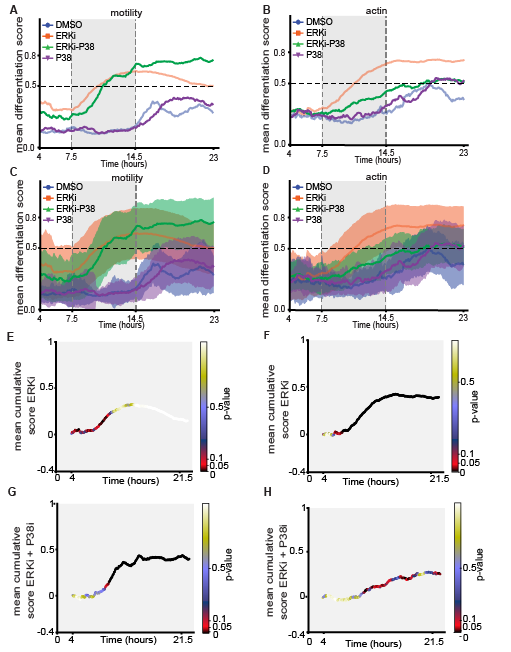


Appendix Figure S14 Differentiation scores for p38i perturbation

**(A-B)** Mean differentiation score over time of ERKi- (orange), DMSO- (blue), ERKi+p38i- (green) and p38i- (purple) treated cells using the motility (A) and actin intensity (B) classifiers. Dashed vertical gray rectangle highlights the differentiation time interval of 7.5-14.5 hours. ERKi- and DMSO- treated cells differentiation scores are the same as shown in Fig. 5A-B. Since co-treated cells undergo differentiation but not fusion, the differentiation score did not decrease after 14.5 hours, unlike ERKi-treated cells that begin to massively fuse at these times and thus change their motility and actin dynamics (ERKi: 575 cells; ERKi+p38i: 208 cells; p38i: 202 cells; DMSO: 103 cells). **(C-D)** Mean differentiation score over time (solid line) and standard deviation (shade) of the cell populations described above (A, B), predicted by the motility (C) and actin intensity (D) classifiers (ERKi: 575 cells; ERKi+p38i: 208 cells; p38i: 202 cells; DMSO: 103 cells). **(E-F)** Average cumulative change in differentiation score over time of ERKi treated cells using the motility (E) and actin intensity models (F). P-values resulting from a t-test between the cumulative change in differentiation score of cells treated with ERKi and ERKi +p38i are color coded. (ERKi: 575 cells; ERKi+p38i: 208 cells). **(G-H)** Average cumulative change in differentiation score over time of Erki+p38i treated cells using the motility (G) and actin intensity models (H). P-values resulting from a t-test between the cumulative change in differentiation score of cells treated with Erki+p38i and ERKi are color coded. (ERKi: 575 cells; ERKi+p38i: 208 cells).

Appendix Figure S15

**
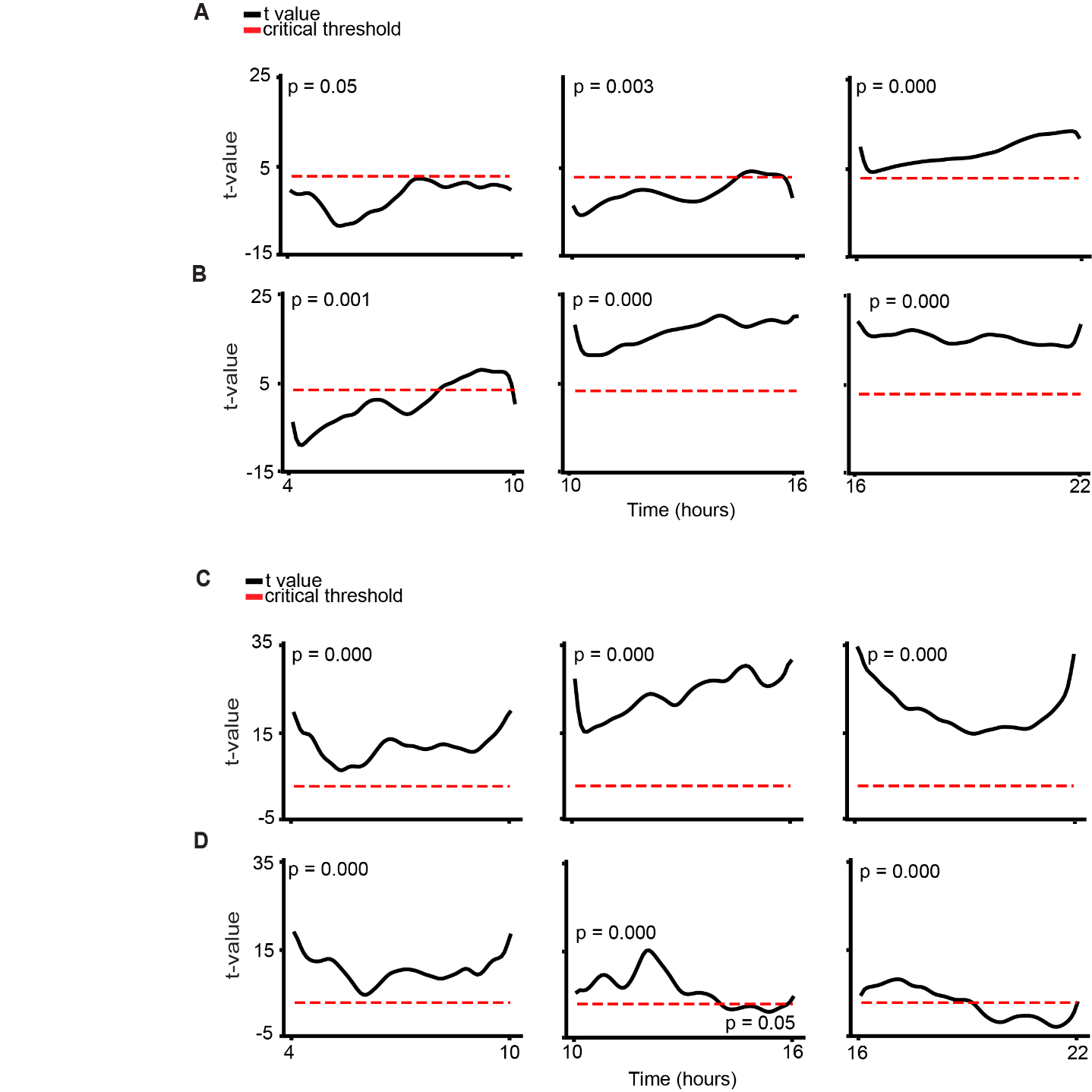
**

Appendix Figure S15 Random Field Theory (RFT) statistical analysis

**(A-B)** RFT analysis of the differences in differentiation scores between cells treated with ERKi + p38i and cells treated with ERKi, using the motility (A) and actin intensity (B) models (ERKi + p38i: 208 cells; ERKi: 575 cells). The black line represents the results of a two-sample t-test conducted over the duration of the experiment. Red dotted horizontal lines indicate the critical RFT threshold at α=0.05. The depicted p-values denote the probability that trajectories within those temporal extents would surpass the critical RFT threshold (refer to Methods for details). A statistically significant difference in differentiation scores is observed during the time intervals of 10-22 hours (motility) and at all time intervals (actin intensity). (C-D) Random Field Theory (RFT) analysis of the differences in differentiation scores between cells treated with ERKi + p38i and cells treated with p38i, using the motility (C) and actin intensity (D) models (ERKi + p38i: 208 cells; p38i: 202 cells). The black line represents the results of a two-sample t-test conducted over the duration of the experiment. Red dotted horizontal lines indicate the critical RFT threshold at α=0.05. The depicted p-values denote the probability that trajectories within those temporal extents would surpass the critical RFT threshold (refer to Methods for details). A statistically significant difference in differentiation scores is observed at all time intervals (motility), and at the time intervals of 0-14, 16-22 hours (actin intensity).

Appendix Figure S16


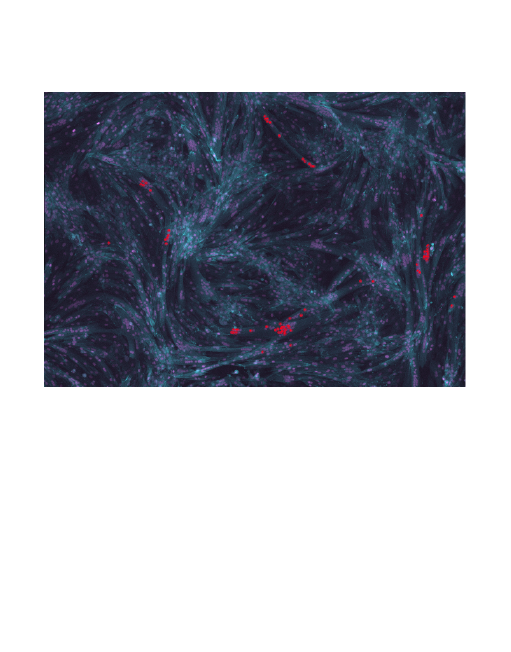


Appendix Figure S16 Nuclei fusion manual annotation

Overlay image showing nuclei in fibers, which were selected for backtracking. All nuclei (magenta) and actin (cyan) are shown.

Appendix Figure S17


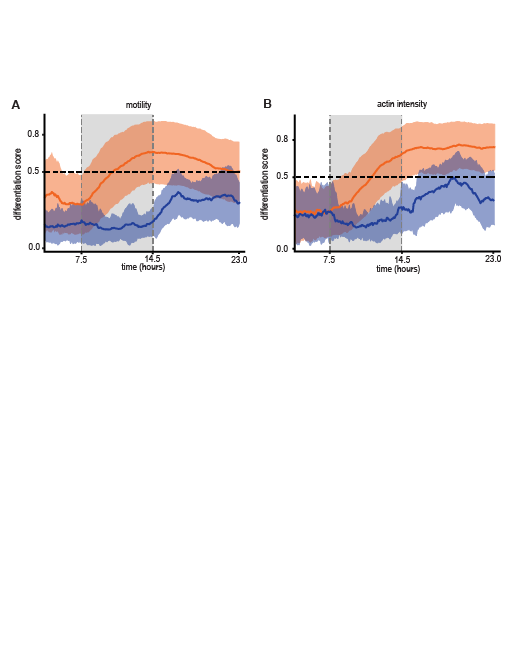


Appendix Figure S17 Differentiation scores over time for the entire experiment

**(A-B)** Mean (solid line) and standard deviation (shade) of the differentiation score over time of ERKi- (orange) and DMSO- (blue) treated cells using the motility (A) and the actin intensity (B) classifiers (ERK: 575 cells; DMSO: 103 cells). Dashed vertical gray rectangle highlights the time interval of 7.5-14.5 hours, where both models predicted the differentiation occurs. The increase of the untreated cells’ differentiation score in concurrence with unchanged (actin) or reduced (motility) scores for ERKi-treated cells around 14.5 hours could be due to altered motility/actin dynamics of untreated cells in denser microenvironments and due to differentiated ERKi-treated cells undergoing fusion.
