# Supplementary material for: Machine learning inference of continuous single-cell state transitions during myoblast differentiation and fusion
Source: Mol Syst Biol. 2024 Jan 18;20(3):217–41. doi: 10.1038/s44320-024-00010-3 (PMC10912675; doi:10.1038/s44320-024-00010-3)
Supplement: Supplementary file 6 — Expanded View Figures [file 44320_2024_10_MOESM6_ESM.pdf]

Expanded View Figures

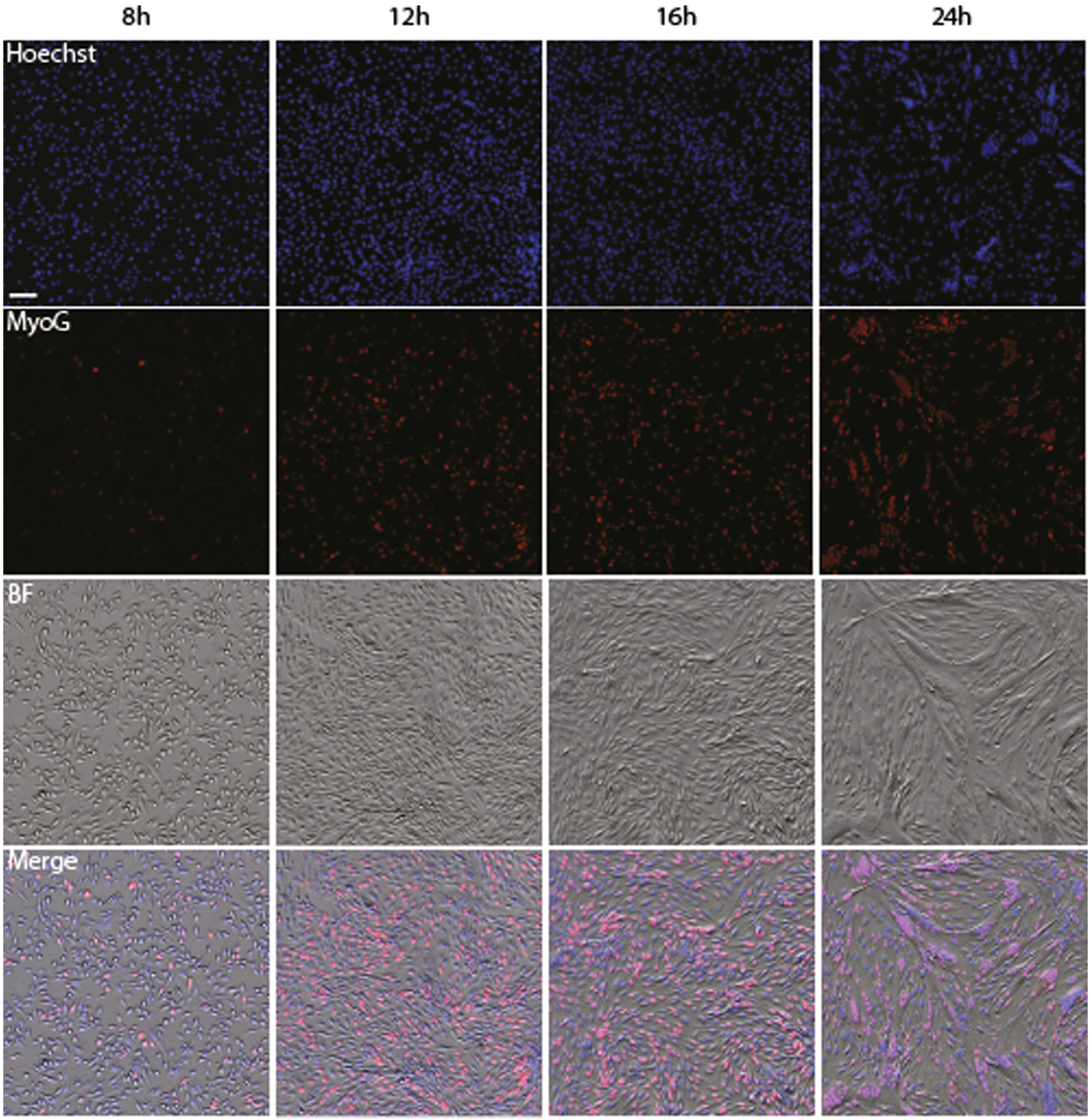

Figure EV1. The number of differentiating myoblasts MyoG-expressing increase over time.

Images of primary myoblasts fixed at different time points after ERK inhibition and stained for nuclei (Hoechst, blue) and MyoG (red), along with Brightfield (gray) and Merge for reference. Magnification ×5. Scale bar 100 μm.

© The Author(s)

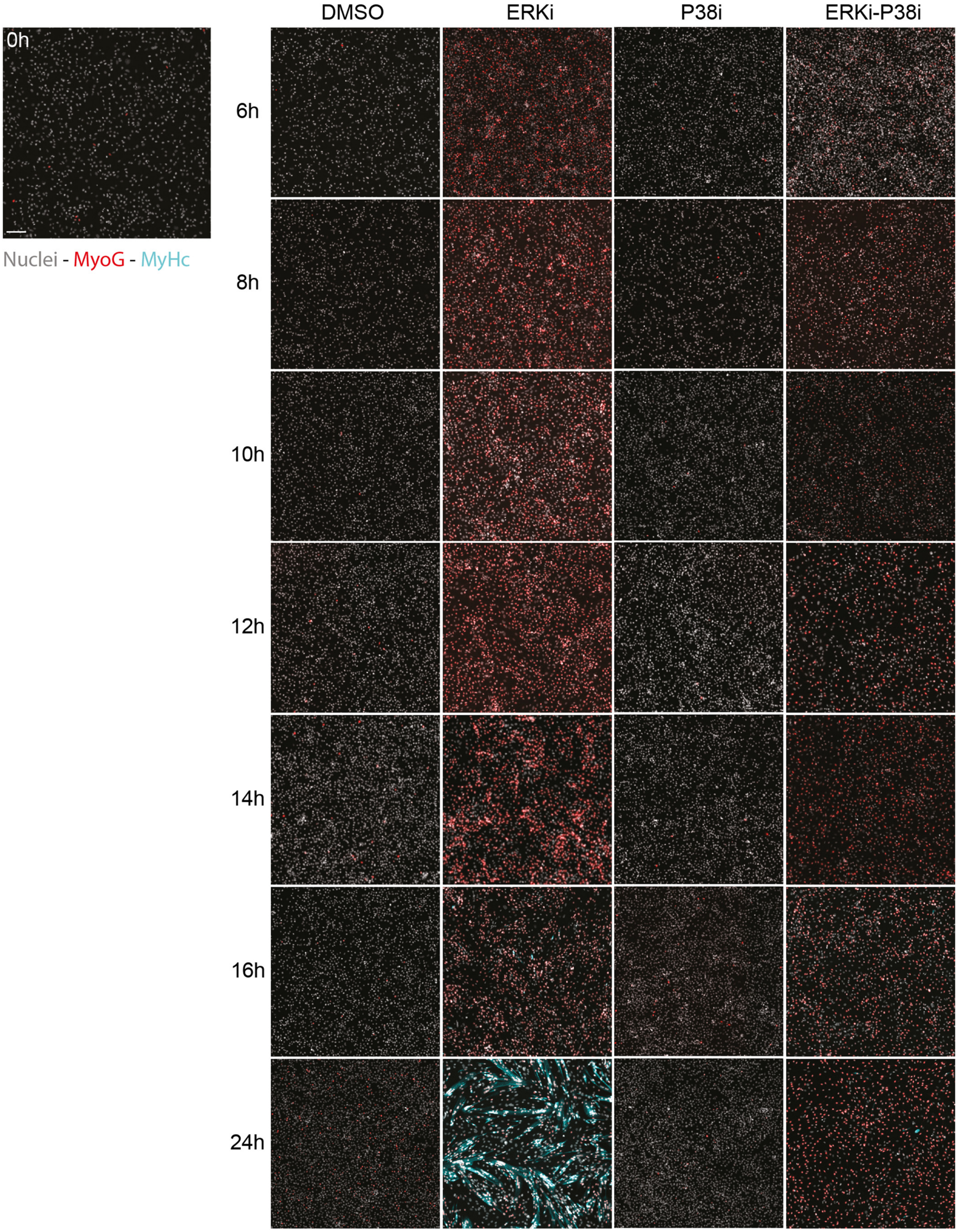
0h
Nuclei - MyoG - MyHc
DMSO
ERKi
P38i
ERKi-P38i
6h
8h
10h
12h
14h
16h
24h

Figure EV2. Immunofluorescence staining of MyoG and MyHC.

Representative immunofluorescence (IF) images of myoblasts at 0, 6, 8, 10, 12, 14, 16, 24 h after treatment with DMSO, p38i 5 μM or ERKi 1 μM or the combination of ERKi-p38i. Cells were stained using anti-MyoG (red), anti-MyHC (cyan), and the nuclear dye Hoechst 33342 (gray). Magnification ×5. Scale bar: 100 μm.

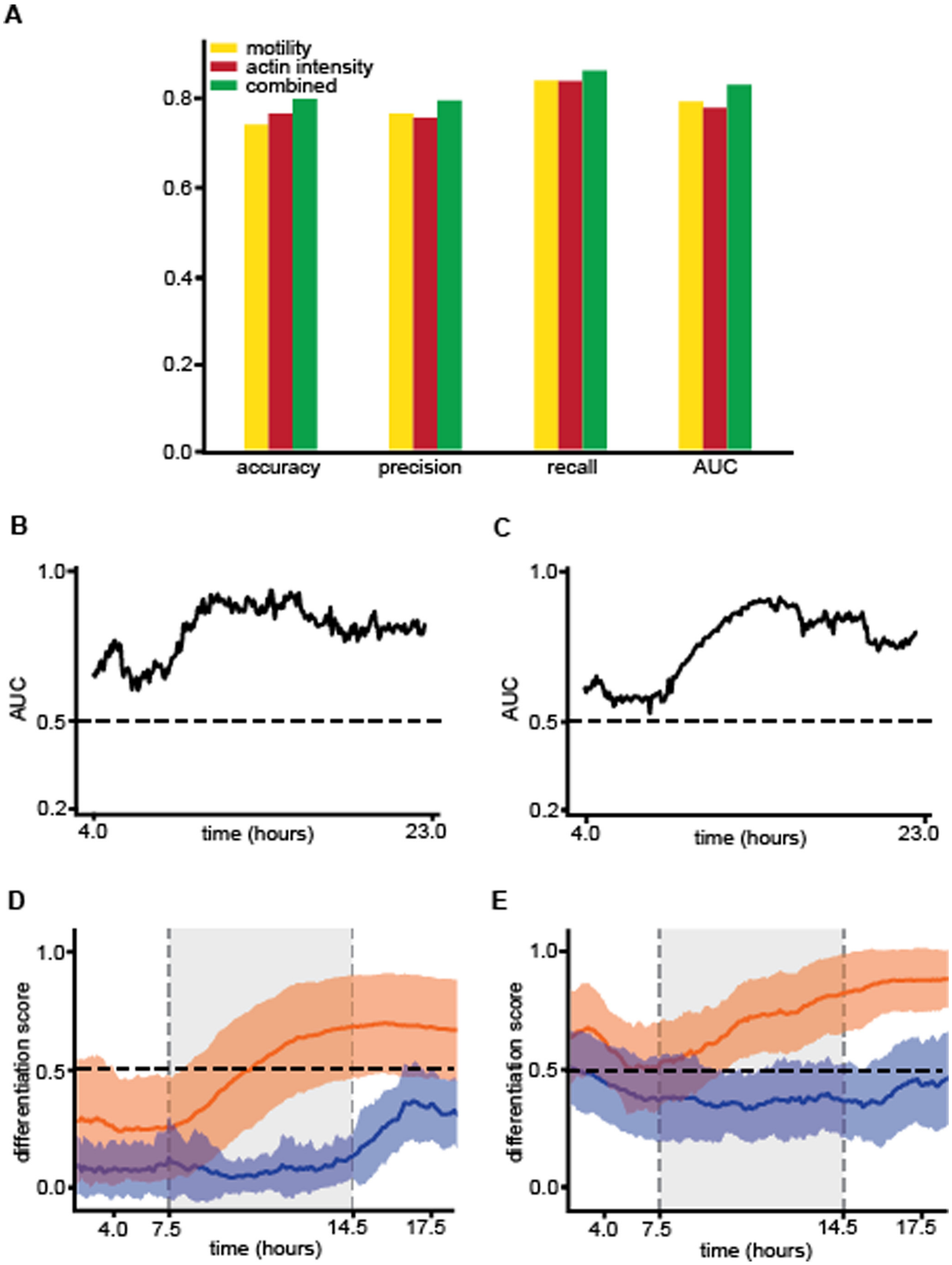
A
motility
actin intensity
combined
0.0
0.2
0.4
0.6
0.8
accuracy
precision
recall
AUC
B
1.0
0.5
0.2
AUC
4.0
time (hours)
23.0
C
1.0
0.5
0.2
AUC
4.0
time (hours)
23.0
D
1.0
0.5
0.0
differentiation score
4.0
7.5
14.5
17.5
time (hours)
E
1.0
0.5
0.0
differentiation score
4.0
7.5
14.5
17.5
time (hours)

 © The Author(s)

◀ **Figure EV3. Performance analysis of a classifier trained on both motility and actin dynamics.**

(**A**) Accuracy, precision, recall and area under the receiver operating characteristic (ROC) curve (AUC) for classifiers trained with motility (yellow), actin intensity (red) and a combination of motility and actin intensity (green) time series. Average accuracy rates were 0.74, 0.77 and 0.8; average precision rates were 0.77, 0.76, and 0.8; Average recall rates were 0.84, 0.84 and 0.87; average AUC rates were 0.8, 0.78 and 0.84 correspondingly. All metrics were calculated for 678 cells from an independent experiment. Overall, the combined classifier exhibits better classification performance. (**B**) Area under the receiver operating characteristic (ROC) curve (AUC) over time for a combined model (N=678 cells). (**C**) AUC over time for a combined model- flipped experiments for train/test (848 cells). (**D**) Mean (solid line) and standard deviation (shade) of the differentiation score over time of ERKi- (orange) and DMSO- (blue) treated cells using the combined classifier. Dashed vertical gray rectangle highlights the time interval of 7.5–14.5 h, where the model predicted the differentiation occurs (ERK: 575 cells; DMSO: 103 cells). (**E**) Mean (solid line) and standard deviation (shade) of the differentiation score over time of ERKi- (orange) and DMSO- (blue) treated cells using the combined classifier-flipped experiments for train/test (ERK: 538 cells; DMSO: 310 cells).

© The Author(s)

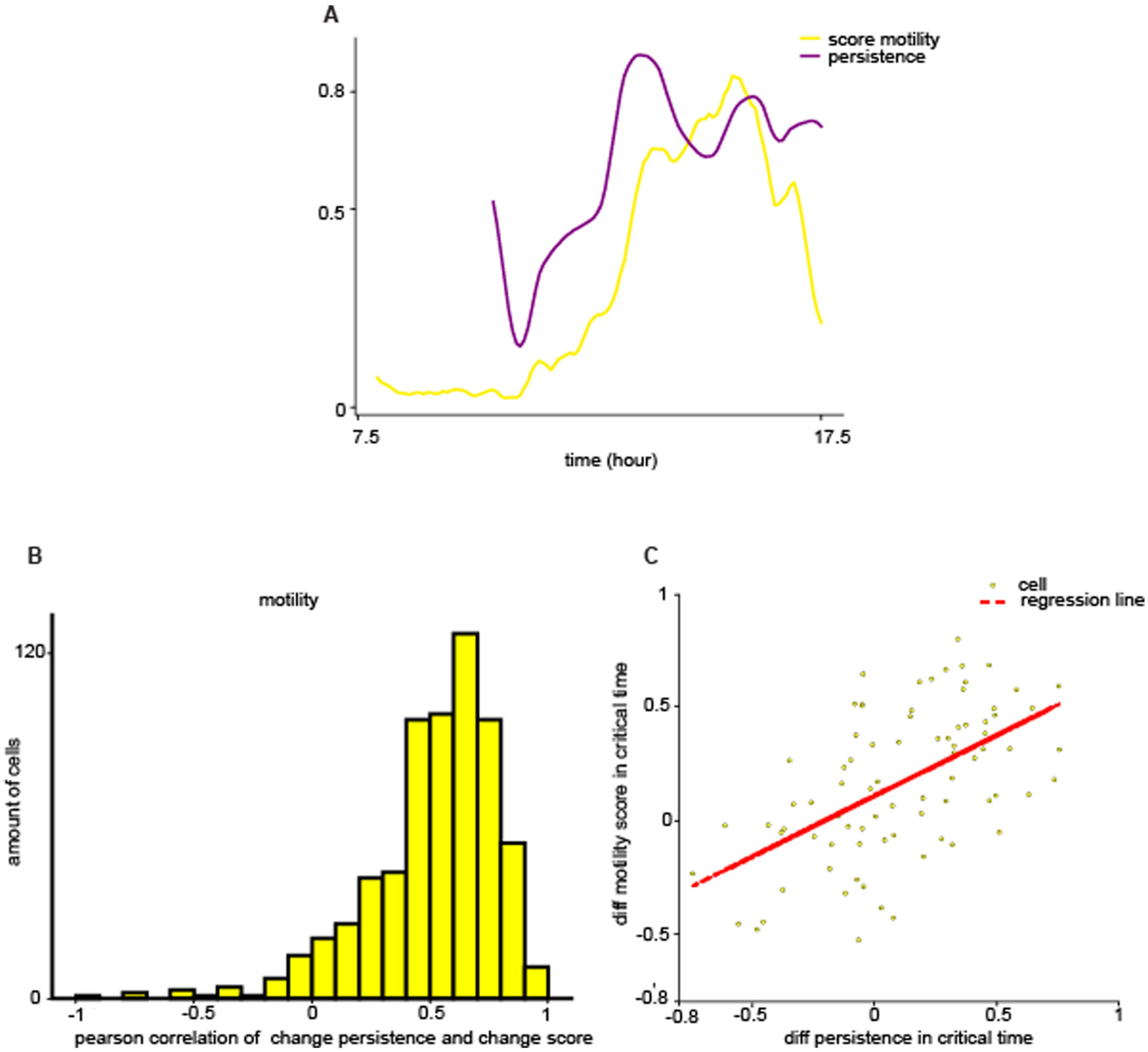

Figure EV4. Persistence in migration is associated with differentiation.

(**A**) Representative single cell's differentiation score (yellow) as predicted by the motility-based model, and persistence in migration rate (purple) through time. (**B**) Distribution of single cells Pearson correlation between the difference in differentiation scores and the difference in persistence rates. Values were calculated within time intervals of 50 min. Mean Pearson correlation coefficient was 0.51. 88.07% of cells showed significant Pearson correlation (Pearson correlation P value < 0.05; $N = 575$ cells). (**C**) Single cells difference in differentiation scores (y axis) over difference in the persistence rate (x axis) between the beginning and end of the critical time window where we identified differentiation occurs (7.5–14.5 h). Red diagonal line indicates the regression line. Pearson correlation coefficient was 0.55 (Pearson correlation P value < 0.0001).

 © The Author(s)

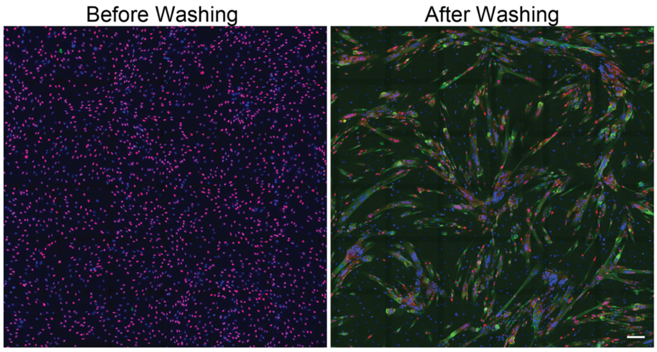

Figure EV5. Co-inhibition of p38 and ERK1/2 leads to properly differentiated ready to fuse cells.

Immunofluorescence images of primary myoblasts treated with p38i+ERKi for 24 h, before and 24 h after the inhibitors were washed. The cells were fixed and stained for MyoG (red), MyHC (green), and the nuclei (Hoechst, blue), scale bar 100 μm. 24 h after treatment with p38i+ERKi, most of the cells expressed MyoG, were MyHC negative, and did not undergo fusion. Thus, the cells were differentiated but unfused. 24 h after the inhibitors were washed, there was a decrease in MyoG-positive cells, and most cells express MyHC, indicating that cells completed the differentiation process and fused.
